# Cutting weight, gaining stress: the hidden battle that outweighs fight day tension in kickboxing

**DOI:** 10.3389/fpsyg.2026.1762659

**Published:** 2026-03-16

**Authors:** Burak Karababa, Süleyman Ulupınar, Cebrail Gençoǧlu, Selim Asan, Deniz Bedir, İzzet İnce, Salih Çabuk, Ferhat Canyurt, Serhat Özbay, Kaan Kaya

**Affiliations:** 1 Department of Physical Education and Sports, Faculty of Sport Sciences, Erzurum Technical University, Erzurum, Türkiye; 2 Department of Coaching Education, Faculty of Sport Sciences, Erzurum Technical University, Erzurum, Türkiye; 3 Department of Coaching Education, Faculty of Sport Sciences, Ankara Yildirim Beyazit University, Ankara, Türkiye; 4 Faculty of Sports Sciences, Istanbul Yeni Yuzyil University, Istanbul, Türkiye

**Keywords:** elite kickboxers, heart rate variability, rapid weight loss, RMSSD, weight-cutting

## Abstract

**Background:**

This study investigated the effects of rapid weight-cutting on stress in elite kickboxers by analyzing Heart Rate Variability (HRV) and subjective stress levels using a Visual Analog Scale (VAS).

**Methods:**

Data were collected across seven standardized time points: Weigh-in Morning, Pre-Weigh-in, Post-Weigh-in, Post–Weigh-in Meal, Match Day Morning, Pre-Match, and Post-Match. Participants were divided into a weight-cutting group (*n* = 12), who reduced at least 5% of their body weight within 48 h before competition, and a control group (*n* = 13), who maintained regular training and nutrition. HRV was measured using a chest-strap electrocardiography-based heart rate sensor (Polar H10) to obtain high-accuracy R–R interval data under real-world competition conditions.

**Results:**

The weight-cutting group showed consistently lower RMSSD values, indicating sustained physiological stress. Subjective stress levels were also higher in this group, peaking before the weigh-in and exceeding pre-match levels. A marked reduction in perceived stress was observed immediately after the weigh-in, suggesting psychological relief. However, despite post-weigh-in rehydration and feeding, RMSSD values remained suppressed on match day, indicating incomplete physiological recovery despite stabilized subjective stress. The control group demonstrated relatively stable HRV and VAS values across all time points.

**Conclusions:**

These findings highlight the prolonged autonomic strain associated with rapid weight-cutting and demonstrate a dissociation between subjective and physiological recovery. Monitoring both autonomic and perceptual stress responses may be essential for safeguarding athlete health and optimizing performance in weight-category sports.

**Trial registration:**

ClinicalTrials.gov, identifier: NCT06860204.

## Introduction

Kickboxing is increasingly popular worldwide as a combat sport (Gençoglu et al., 2023; Podrigalo et al., 2022). Competitions in this sport are organized according to weight categories to maintain physical equity among athletes (Ouergui et al., 2014; Hoelbling et al., 2023). Athletes commonly engage in losing weight before competitions to qualify for lighter weight classes; this process is widely known as “weight cutting” (Franchini et al., 2012; Barley et al., 2019; Crighton et al., 2016). It involves athletes temporarily reducing their body mass to meet a category requirement, with most of the lost weight typically regained rapidly after the weigh-in (Zhong et al., 2024; Dugonjić et al., 2019; Artioli et al., 2016). This practice is often assumed to provide a potential competitive advantage, as it may allow an athlete to compete with a larger body mass than their opponents; however, the extent of this advantage appears to be highly context-dependent. However, the physiological and psychological impacts of this practice yield mixed results in sports science literature (Artioli et al., 2016; Burke et al., 2021; Lakicevic et al., 2021).

The weight-cutting process can have considerable effects on athletes' physiological and psychological health. Physiologically, it often leads to reductions in muscle mass, dehydration, and electrolyte imbalance (Crighton et al., 2016; Lakicevic et al., 2021; Durguerian et al., 2016). For instance, a study by Barley and colleagues demonstrated that the majority of combat sport athletes lose approximately 5% of their body weight within the 24 h preceding a competition, which adversely affects anaerobic capacity and the ability to perform repeated high-intensity efforts (Barley et al., 2018). Psychologically, weight cutting can induce stress, anxiety, and decreased motivation. Previous studies have reported that a significant majority (up to 91%) of athletes experience negative psychological effects during weight cutting, including fatigue, anger, feelings of isolation, and anxiety (Kwan and Helms, 2022; Franchini et al., 2012; Levy and Boyd, 2025).

Further emphasizing the prevalence and severity of this practice, a study by Boris Dugonjić and colleagues highlighted the universality of weight-cutting among elite kickboxers, revealing that all participating athletes (100%) engage in weight-cutting before competitions (Dugonjić et al., 2019). In this context, elite kickboxers are defined as athletes competing at national or international championship level with several years of competitive experience. Most kickboxers typically lose between 2 and 5% of their body mass, with approximately 30% losing between 6 and 8%. Alarmingly, nearly 30% reported cutting 10% or more of their body weight at least once in their careers. These findings underscore the widespread nature of weight-cutting practices in elite kickboxing and highlight their potential implications for physical performance and psychological wellbeing (Dugonjić et al., 2019).

The development of wearable technologies has facilitated continuous health monitoring for combat sports athletes, enabling real-time measurement of critical physiological parameters, such as heart rate variability (HRV) (Kim et al., 2018; Schaffarczyk et al., 2022). HRV reflects the variability in milliseconds between heartbeats, offering key insights into overall health, stress levels, and fatigue by indicating the balance between the sympathetic and parasympathetic nervous systems (Shaffer and Ginsberg, 2017; Bourdillon et al., 2022). Root Mean Square of the Successive Differences (RMSSD), a commonly used HRV metric, represents the square root of the mean squared differences between successive heartbeats and is widely recognized as an indicator of parasympathetic nervous system activity. While such physiological data are crucial for understanding autonomic stress responses, integrating subjective assessments can further enhance this understanding. Subjective evaluations, such as the Visual Analogue Scale (VAS) for stress, offer insight into perceived stress and recovery, highlighting potential discrepancies between measured physiological stress and the athlete's felt experience, which could impact performance and wellbeing. Although previous studies have examined either physiological or psychological responses to weight cutting, evidence jointly monitoring objective autonomic markers and subjective stress perceptions under real competition conditions across multiple, closely spaced time points remains limited, particularly in elite kickboxing. In addition, few studies have captured stress dynamics across the entire competition week, including transitional phases such as the post–weigh-in meal period. The aim of this study is to investigate the effects of the weight reduction process on both objective (RMSSD measurements) and subjective (VAS) stress parameters in kickboxers across seven specific time points: Morning of Weigh-in, Pre-Weigh-in, Post-Weigh-in, Post-Weigh-in Meal, Morning of Match Day, Pre-Match, and Post-Match.

## Method

### Research design

This study employed an observational comparative design to examine objective and subjective stress responses associated with the weight-cutting process in elite kickboxers. Conducted under real competition conditions during the Senior European Kickboxing Championship (All Categories) in Greece from November 2 to November 10, 2024, the study involved two groups: a weight-cutting group and a non-weight-cutting group. This design allowed for dual comparisons: between objective (RMSSD) and subjective (VAS) stress measures, as well as between athletes who engaged in weight reduction and those who did not. Data were collected at seven key time points—Weigh-in Morning, Pre-Weigh-in, Post-Weigh-in, Post–Weigh-in Meal, Match Day Morning, Pre-Match, and Post-Match—to capture stress fluctuations across critical stages of the weight-cutting and competition period.

### Participants

The study included elite kickboxers selected based on specific inclusion criteria to ensure relevance and consistency in data. Using G^*^Power analysis, the required sample size was calculated to achieve sufficient statistical power for the study's objectives. This analysis, conducted with an ANOVA: Repeated measures, within-between interaction model (Faul et al., 2007), indicated a minimum of 22 participants, which would maintain the study's statistical strength. The parameters for the sample size calculation were as follows: an effect size (*f* ) of 0.25, representing a medium effect size; an alpha level (α) of 0.05, allowing for a 5% probability of Type I error; and a power (1 – β) of 0.90, giving a 90% chance of detecting a true effect. The analysis accounted for two groups (weight-cutting and non-weight-cutting athletes) and seven measurements at different time intervals. Despite the G^*^Power calculation, we set an initial target of 15 participants per group to account for potential data loss. Accordingly, the study was initiated with 30 elite kickboxers. However, five athletes were unable to complete the full measurement protocol due to competition-week constraints, including scheduling conflicts and logistical challenges inherent to real-world tournament settings. The study was ultimately completed with 25 participants, including 12 in the weight-cutting group and 13 in the non-weight-cutting group.

Participants were active, elite male kickboxers aged between 18 and 35, each with at least 5 years of competitive experience and active participation at national or international championship level, consistent with the operational definition of elite athletes used in the Introduction. Athletes in the weight-cutting group were defined as those who had lost at least 5% of their body weight within the 48 h prior to weigh-in. Conversely, participants in the non-weight-cutting group did not engage in any weight-cutting procedures. This group only experienced a minimal weight reduction, not exceeding 2% of their body mass, during their final training session before the weigh-in and followed their regular nutrition routine, including post-weigh-in meals, without any calorie restrictions. Although minimal, this degree of body mass fluctuation may still influence physiological stress responses and is therefore acknowledged as a potential methodological limitation. To maintain clear group separation, athletes who lost between approximately 3 and 4% of their body mass were predefined as an exclusion criterion and were not included in the analyses. Accordingly, the control group consisted of athletes who maintained a routine diet and training regimen without deliberate weight reduction.

Participation in the study was entirely voluntary. Each athlete was provided with detailed information about the study's purpose, methodology, and potential risks, and written informed consent was obtained. Participants were assured of their right to withdraw from the study at any point, with the understanding that their data would not be used if they chose to do so. Confidentiality of personal data was strictly maintained, and all information was used solely for research purposes. This study was approved by the Erzurum Technical University Scientific Research and Publication Ethics Committee (Meeting No: 10, Decision No: 10, Date: 12.09.2024).

### Data collection

Data collection focused on both objective and subjective stress parameters, measured using HRV and the VAS, respectively. Objective stress data were gathered through RMSSD values, recorded using a chest-strap electrocardiography-based heart rate sensor (Polar H10), which provides high-accuracy R–R interval data suitable for HRV analysis. This device detects cardiac electrical activity via skin-contact electrodes rather than photoplethysmography, offering ECG-comparable precision in identifying successive inter-beat intervals. Before each HRV recording, athletes rested quietly in a seated position for 5 min to stabilize cardiovascular activity. Each participant wore the chest strap in a calm, seated position, with measurements taken over a 5-min period to ensure consistency and reliability of the HRV data. HRV recordings were obtained under spontaneous breathing conditions, without paced or controlled respiration, to reflect real-world competition settings; this approach is acknowledged as a potential limitation given the sensitivity of RMSSD to respiratory patterns. Raw R–R interval files were exported in.txt/.csv format and processed using Kubios HRV (Premium), where artefact correction (Automatic/Medium) and detrending (Smoothness Priors, λ = 500) were applied prior to RMSSD calculation. These preprocessing settings were selected in accordance with the default recommendations of the Kubios HRV software for short-term resting recordings in applied field settings.

Subjective stress data were obtained through the VAS, where participants rated their perceived stress levels on a scale, providing a simple yet effective measure of subjective stress at each designated interval. Prior to the first measurement session, athletes received a brief explanation of the VAS using illustrative examples to ensure proper understanding of the scale. At each measurement point, athletes rated their perceived stress on a 0–10 scale, marked on a 10-cm horizontal line anchored with “no stress at all” (0) on the left end and “extreme stress” (Artioli et al., 2016) on the right end. Participants were instructed to indicate their current overall stress level by marking a single point on the scale, reflecting how they felt at that specific moment. The VAS assessment was administered immediately after the HRV recording at each time point to avoid any potential influence on autonomic measurements. Data were collected at seven specific time points surrounding the weigh-in and competition period: Weigh-in Morning, Pre-Weigh-in, Post-Weigh-in, Post–Weigh-in Meal, Match Day Morning, Pre-Match, and Post-Match.

### Data analysis

Data were analyzed using SPSS version 26. For pairwise comparisons between groups, Hedges' *g* effect size calculations with a correction for small sample sizes were conducted to determine the magnitude of differences. Effect sizes were interpreted based on Hopkins' classification system, which provides a practical framework for understanding the impact of observed differences. According to this classification (Hopkins, 2002), effect sizes are categorized as follows: trivial (< 0.2), small (0.2–0.59), moderate (0.60–1.19), large (1.20–1.99), very large (2.0–3.99), and nearly perfect (> 4.0). To examine changes across the seven time points for both objective (RMSSD) and subjective (VAS) stress measurements, repeated measures ANOVA with a within-between interaction was used. The assumption of sphericity was assessed using Mauchly's test. When the assumption of sphericity was violated, Greenhouse–Geisser corrections were applied to adjust the degrees of freedom. Corrected degrees of freedom and corresponding *p*-values are reported where applicable. When significant main effects or interactions were detected, Bonferroni-adjusted *post hoc* tests were applied to control for Type I error across multiple comparisons. Additionally, partial eta-squared (η^2^_p_) values were reported to assess practical significance, offering insight into the relative importance of the observed effects. Statistical significance was set at *p* < 0.05.

## Results

**Figure 1:**
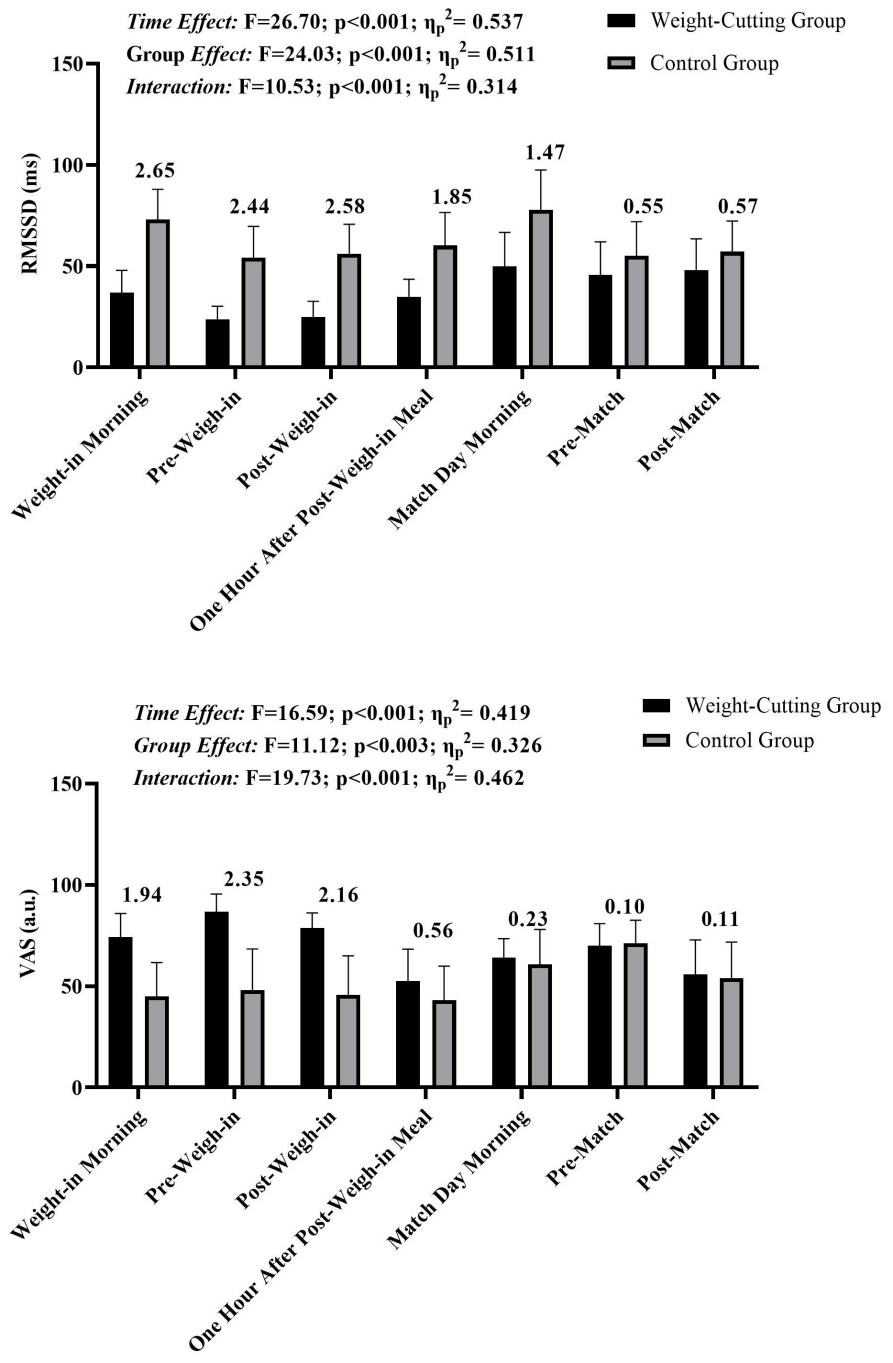
Changes in RMSSD and VAS across seven measurement time points in weight-cutting and control groups. Values are presented as mean ± SD. RMSSD reflects parasympathetic autonomic modulation, while VAS represents perceived stress levels. Black bars indicate the weight-cutting group and grey bars indicate the control group. Results of repeated-measures ANOVA (time effect, group effect, and time × group interaction) are shown above each panel. Numbers above the bars represent Hedges' *g* effect sizes for between-group comparisons at each time point.

The results for RMSSD and VAS measurements across seven time points for the weight-cutting and control groups are presented in Figure 1, Table 1. For RMSSD values, there was a significant main effect of time (*F* = 26.70, *p* < 0.001, η^2^_p_ = 0.537) and a significant main effect of group (*F* = 24.03, *p* < 0.001, η^2^_p_ = 0.511), as well as a significant time-by-group interaction effect (*F* = 10.53, *p* < 0.001, η^2^_p_ = 0.314). The significant group main effect indicates that, when averaged across all time points, RMSSD values were overall lower in the weight-cutting group compared with the control group. The control group consistently displayed higher RMSSD values compared to the weight-cutting group at each time point. Effect size classifications for RMSSD comparisons between groups are indicated above each time point. The control group displayed a “Very Large” effect size in favor of higher RMSSD values during Weight-in Morning, Pre-Weigh-in, and Post-Weigh-in. At the “Post-Weigh-in Meal and Match Day Morning” time points, the effect size was classified as “Large,” and this effect size reduced to “Small” during the Pre-Match and Post-Match time points.

**Table 1:** MSSD and VAS values across seven time points in weight-cutting and control groups.

	**RMSSD**		**VAS**	
	**Weight-cutting**	**Control**	**Weight-cutting**	**Control**
Weigh-in morning	36.92 ± 10.97	73.08 ± 14.94	74.17 ± 11.65	45.01 ± 16.71
Pre-weigh-in	23.83 ± 6.42	54.15 ± 15.47	86.67 ± 8.88	48.08 ± 20.26
Post-weigh-in	24.92 ± 7.73	56.23 ± 14.48	78.75 ± 7.42	45.77 ± 19.24
Post-weigh-in meal	34.92 ± 8.68	60.23 ± 16.33	52.50 ± 15.74	43.08 ± 16.78
Match day morning	50.00 ± 16.64	77.77 ± 19.69	64.17 ± 9.25	60.77 ± 17.30
Pre-match	45.75 ± 16.32	55.15 ± 16.84	70.00 ± 10.87	71.15 ± 11.39
Post-match	48.17 ± 15.34	57.15 ± 15.20	55.83 ± 17.03	53.85 ± 17.93

Values are presented as mean ± SD.

RMSSD, root mean square of successive differences; VAS, visual analog scale.

For VAS measurements, there was also a significant main effect of time (*F* = 16.59, *p* < 0.001, η^2^_p_ = 0.419) and a significant main effect of group (*F* = 11.12, *p* = 0.003, η^2^_p_ = 0.326), in addition to a significant interaction effect (*F* = 19.73, *p* < 0.001, η^2^_p_ = 0.462). The significant group main effect reflects overall higher perceived stress levels in the weight-cutting group compared with the control group across the measurement period. The weight-cutting group showed higher VAS scores than the control group at each time point. Effect size classifications for VAS scores between groups were “Large” at the Weight-in Morning time point and “Very Large” at Pre-Weigh-in and Post-Weigh-in. After the meal following the weigh-in, the effect size decreased to “Small,” remaining at this level until the Pre-Match and Post-Match points, where it was classified as “Trivial” (Figure 1).

In the weight-cutting group, a “Large” decrease in RMSSD was observed from Weigh-in Morning to Pre-Weigh-in. From Pre-Weigh-in to Post-Weigh-in, there was a “Small” increase in RMSSD, despite the athletes not having eaten. Following the post-weigh-in meal, a “Moderate” increase was observed from Post-Weigh-in to Post-Weigh-in Meal. Between Post-Weigh-in Meal and Match Day Morning, an additional “Moderate” increase was observed, suggesting a continued recovery overnight. However, RMSSD showed a “Small” decrease from Match Day Morning to Pre-Match. Finally, from Pre-Match to Post-Match, a “Small” increase was noted, indicating a minor recovery following the match. It is also noteworthy that Weigh-in Morning displayed a “Large” effect size in comparison to Match Day Morning, indicating significantly higher stress levels at the start of weigh-in day than on the morning of the match. Similarly, the Pre-Weigh-in time point showed a “Large” effect size when compared to Pre-Match, highlighting that the period immediately before the weigh-in was substantially more stressful than the period immediately before the match (Table 2).

**Table 2:** Hedges' *g* effect size matrix of RMSSD changes across measurement times for weight cutting group.

	**Pre-weigh-in**	**Post-weigh-in**	**Post-weigh-in meal**	**Match day morning**	**Pre-match**	**Post-match**
Weigh-in morning	1.84	1.60	0.18	−1.38	−0.87	−1.07
Pre-weigh-in		−0.25	−1.22	−1.78	−1.42	−1.61
Post-weigh-in			−0.97	−1.59	−1.30	−1.48
Post-weigh-in meal				−0.87	−0.63	−0.82
Match day morning					0.87	0.20
Pre-match						0.30

Values represent Hedges' g effect sizes calculated for within-group pairwise comparisons following Bonferroni-adjusted post hoc tests. Effect size magnitudes were interpreted according to Hopkins' scale: trivial (< 0.20), small (0.20–0.59), moderate (0.60–1.19), large (1.20–1.99), very large (2.00–3.99), and nearly perfect (≥4.00). Positive and negative values indicate the direction of change between the respective time points.

In the control group, RMSSD values showed a “Very Large” decrease from Weigh-in Morning to Pre-Weigh-in. From Pre-Weigh-in to Post-Weigh-in, a “Small” increase was observed. Subsequently, from Post-Weigh-in to Post-Weigh-in Meal, there was a “Moderate” increase in RMSSD. A continued “Moderate” increase was noted from Post-Weigh-in Meal to Match Day Morning, indicating additional overnight recovery. However, a “Large” decrease was observed from Match Day Morning to Pre-Match, suggesting elevated stress levels as the competition drew near. From Pre-Match to Post-Match, a “Small” increase was recorded. Additionally, a “Small” effect size was observed between Weigh-in Morning and Match Day Morning, indicating a minor difference in RMSSD between these two mornings. In contrast, the difference between Pre-Weigh-in and Pre-Match was “Trivial,” suggesting minimal physiological change between these time points for the control group (Table 3).

**Table 3:** Hedges' *g* effect size matrix of RMSSD changes across measurement times for control group.

	**Pre-weigh-in**	**Post-weigh-in**	**Post-weigh-in meal**	**Match day morning**	**Pre-match**	**Post-match**
Weigh-in morning	2.20	1.72	1.28	−0.51	1.39	1.20
Pre-weigh-in		−0.30	−1.02	−1.74	−0.06	−0.19
Post-weigh-in			−0.87	−1.46	0.07	−0.06
Post-weigh-in meal				−1.12	0.29	0.18
Match day morning					1.86	1.42
Pre-match						−0.28

Values represent Hedges' g effect sizes calculated for within-group pairwise comparisons following Bonferroni-adjusted post hoc tests. Effect size magnitudes were interpreted according to Hopkins' scale: trivial (< 0.20), small (0.20–0.59), moderate (0.60–1.19), large (1.20–1.99), very large (2.00–3.99), and nearly perfect (≥4.00). Positive and negative values indicate the direction of change between the respective time points.

In the weight-cutting group, VAS values exhibited a “Very Large” increase from Weigh-in Morning to Pre-Weigh-in. Following the weigh-in, a “Large” decrease was observed from Pre-Weigh-in to Post-Weigh-in, even though the athletes had not yet eaten, suggesting an immediate reduction in perceived stress after completing the weigh-in. This “Large” decrease continued from Post-Weigh-in to Post-Weigh-in Meal. From Post-Weigh-in Meal to Match Day Morning, a “Moderate” decrease in VAS was observed, reflecting continued recovery overnight. However, from Match Day Morning to Pre-Match, there was a “Moderate” increase, suggesting rising stress levels as the competition approached. Finally, from Pre-Match to Post-Match, another “Moderate” decrease in VAS was observed. Notably, Weigh-in Morning was rated as “Moderate” levels more stressful than Match Day Morning, while Pre-Weigh-in was “Large” levels more stressful than Pre-Match, highlighting the heightened stress athletes experience before the weigh-in compared to pre-competition (Table 4).

**Table 4:** Hedges' *g* effect size matrix of VAS changes across measurement times for weight cutting group.

	**Pre-weigh-in**	**Post-weigh-in**	**Post-weigh-in meal**	**Match day morning**	**Pre-Match**	**Post-match**
Weigh-in morning	−2.01	−0.73	1.32	0.98	0.28	0.94
Pre-weigh-in		1.86	2.09	2.89	1.37	1.60
Post-weigh-in			1.74	2.19	0.79	1.28
Post-weigh-in meal				−0.74	−0.99	−0.17
Match day morning					−0.86	0.60
Pre-match						1.10

Values represent Hedges' g effect sizes calculated for within-group pairwise comparisons following Bonferroni-adjusted post hoc tests. Effect size magnitudes were interpreted according to Hopkins' scale: trivial (< 0.20), small (0.20–0.59), moderate (0.60–1.19), large (1.20–1.99), very large (2.00–3.99), and nearly perfect (≥4.00). Positive and negative values indicate the direction of change between the respective time points.

In the control group, VAS values showed a “Small” increase from Weigh-in Morning to Pre-Weigh-in. Following the weigh-in, a “Small” decrease was observed from Pre-Weigh-in to Post-Weigh-in, despite athletes not having eaten, suggesting a minor reduction in perceived stress immediately after the weigh-in. Another “Small” decrease occurred from Post-Weigh-in to Post-Weigh-in Meal. A “Moderate” decrease was noted from Post-Weigh-in Meal to Match Day Morning, indicating further recovery overnight. However, from Match Day Morning to Pre-Match, there was a “Moderate” increase in VAS, reflecting heightened stress as the competition neared. Finally, from Pre-Match to Post-Match, a “Moderate” decrease in VAS was observed, suggesting some relief in perceived stress after the competition concluded. In contrast to the weight-cutting group, Match Day Morning was rated as “Large” levels more stressful than Weigh-in Morning in the control group, and Pre-Match was “Large” levels more stressful than Pre-Weigh-in (Table 5).

**Table 5:** Hedges' *g* effect size matrix of VAS changes across measurement times for control group.

	**Pre-weigh-in**	**Post-weigh-in**	**Post-weigh-in meal**	**Match day morning**	**Pre-match**	**Post-match**
Weigh-in morning	−0.36	−0.08	0.18	−1.55	−2.02	−0.61
Pre-weigh-in		0.38	0.43	−0.80	−1.31	−0.28
Post-weigh-in			0.33	−0.85	−1.40	−0.35
Post-weigh-in meal				−1.11	−1.83	−0.52
Match day morning					−0.81	0.81
Pre-match						1.60

Values represent Hedges' g effect sizes calculated for within-group pairwise comparisons following Bonferroni-adjusted post hoc tests. Effect size magnitudes were interpreted according to Hopkins' scale: trivial (< 0.20), small (0.20–0.59), moderate (0.60–1.19), large (1.20–1.99), very large (2.00–3.99), and nearly perfect (≥4.00). Positive and negative values indicate the direction of change between the respective time points.

## Discussion

This study aimed to examine the effects of the weight-cutting process on objective (RMSSD) and subjective (VAS) stress parameters in kickboxers, providing insights into how this challenging process impacts both physiological and psychological stress levels in athletes. The findings revealed that athletes in the weight-cutting group experienced significant stress starting from the morning of the weigh-in day, with elevated levels persisting through the weigh-in and extending into match day. Although the weight-cutting group consistently exhibited lower RMSSD values compared to the control group at each time point, indicating higher physiological stress, VAS scores were generally aligned with RMSSD values except on match day. On match day, the weight-cutting group reported subjective stress levels similar to those of the control group, suggesting they felt comparably recovered; however, RMSSD values indicated that they had not fully recovered physiologically. This dissociation highlights the potential mismatch between perceived recovery and underlying autonomic regulation. Additionally, pre-weigh-in stress levels in the weight-cutting group surpassed even those observed pre-match, underscoring the intense strain associated with the weight-cutting process. Additionally, pre-weigh-in stress levels in the weight-cutting group surpassed even those observed pre-match, underscoring the intense strain associated with the weight-cutting process. These findings suggest that while the weight-cutting group might feel better subjectively as competition approaches, the physiological toll of weight reduction lingers, contrasting with the more stable responses seen in the control group, which maintained balanced stress markers throughout the pre-competition period.

The physiological impact of weight-cutting on autonomic regulation is evident in the significant decrease in RMSSD values observed in the weight-cutting group, reflecting reduced parasympathetic (vagal) modulation rather than a direct increase in sympathetic activity. RMSSD is widely recognized as a marker predominantly sensitive to cardiac vagal control, and decreases in this parameter are commonly interpreted as vagal withdrawal. However, HRV responses are inherently multifactorial and may also be influenced by factors such as hydration status, sleep quality, psychological arousal, and competition-related stress, all of which may interact during the weigh-in and match period. This reduction in RMSSD aligns with previous findings in combat sports, where athletes undergoing rapid weight loss show substantial stress imposed on the body's autonomic regulatory systems. For example, previous studies have highlighted that weight-cutting processes can lead to reduced recovery capacity and increased physiological strain (Barley et al., 2019; Kwan and Helms, 2022; Franchini et al., 2012; Levy and Boyd, 2025). In particular, Barley et al. (2019) work demonstrated that combat athletes who experienced a 5% body weight reduction prior to competition exhibited notable declines in anaerobic performance and cardiovascular stability, outcomes that are consistent with the observed suppression of vagal modulation reflected by reduced RMSSD values in the weight-cutting group. Such interpretations are consistent with established HRV guidelines, which emphasize that RMSSD primarily reflects parasympathetic cardiac modulation rather than sympathetic drive (Franchini et al., 2012; Levy and Boyd, 2025; Morales et al., 2014).

Additionally, the consistent difference in RMSSD between the weight-cutting and control groups at each time point, with lower values in the weight-cutting group, suggests that the physiological toll of rapid weight loss may persist beyond the initial dehydration and caloric restriction phases. This finding extends the observations by Dugonjić et al. (2019), who reported similar autonomic disruptions in elite kickboxers due to frequent and intense weight-cutting practices. The fact that RMSSD levels in our weight-cutting group showed only modest recovery after rehydration and food intake reinforces the notion that rapid weight loss exerts a prolonged impact on autonomic balance, an effect that could potentially impair an athlete's performance and increase the risk of cardiovascular strain. This reduction in RMSSD during the critical phases of weigh-in and match day suggests that the autonomic stress associated with weight cutting may place athletes at a disadvantage, as the parasympathetic system is crucial for promoting recovery, controlling inflammatory responses, and stabilizing heart rate. These findings align with research indicating that athletes undergoing repeated weight cuts exhibit a compromised physiological state leading into competition, which may affect their ability to achieve peak performance when it is most needed.

Previous research reported that rapid weight loss in combat athletes leads to significant increases in tension, confusion, and fatigue, alongside reduced vigor in the days prior to competition (Dugonjić et al., 2019; Artioli et al., 2016; Koral and Dosseville, 2009). Such psychological strain is further corroborated by various researchers who found that athletes engaged in weight-cutting practices often experience heightened emotional responses, including anger, fatigue, and even depression (Franchini et al., 2012; Zhong et al., 2024; Dugonjić et al., 2019; Lakicevic et al., 2020). These responses can impact mental clarity, focus, and overall mood, potentially reducing the athlete's ability to perform optimally under competitive pressure. In our study, while the weight-cutting group exhibited higher VAS scores than the control group in most pre-competition time points—particularly before the weigh-in—the two groups showed similar VAS scores on match day. This convergence in subjective stress levels suggests that weight-cutting athletes may perceive a sense of recovery by the time of competition, feeling comparably prepared to their non-weight-cutting peers. However, despite this subjective improvement, physiological data (RMSSD) indicated that weight-cutting athletes had not yet fully recovered, revealing a lingering autonomic strain not perceived by the athletes themselves. This pattern underscores the gap between perceived and physiological recovery following weight-cutting, suggesting that subjective assessments alone may not accurately capture the enduring stress effects of weight reduction. Moreover, these elevated pre-weigh-in stress markers in the weight-cutting group—consistent with symptoms observed in other studies, such as low self-esteem, anxiety, and concentration issues—raise concerns about the psychological impact of weight-cutting on athletic performance (Franchini et al., 2012). Continuous exposure to such stressors could increase susceptibility to long-term psychological effects, including mood and eating disorders (Franchini et al., 2012; Zhong et al., 2024; Artioli et al., 2016).

In the present study, the weight-cutting group displayed lower RMSSD values and higher VAS scores than the control group, underscoring the elevated physiological and psychological strain associated with rapid weight reduction. Significant fluctuations were observed across time points for both groups; however, the weight-cutting group demonstrated particularly intense stress responses at critical moments, with weigh-in stress surpassing even the pre-match stress levels. A notable finding was the “large” decrease in subjective stress observed immediately after the weigh-in, despite the athletes not yet eating. This suggests that simply gaining the freedom to eat provided substantial psychological relief, underscoring the impact of perceived restrictions on subjective wellbeing. This finding is consistent with the research by Franchini et al. (2012) and Burke et al. (2021), which highlight that combat sports athletes who engage in weight-cutting often experience substantial autonomic stress and heightened perceived exertion levels (Burke et al., 2021). These studies suggest that while weight-cutting may offer theoretical competitive advantages by enabling athletes to compete in lower weight classes, the resulting stress can detrimentally impact recovery and overall performance.

On match day, although the weight-cutting group reported feeling subjectively similar to the control group (as reflected in VAS scores close to control levels during the morning, pre-match, and post-match), RMSSD values revealed that they had not fully recovered physiologically. This discrepancy between subjective recovery and objective autonomic markers highlights the enduring impact of the weight-cutting process on the body's stress response system, even after athletes feel they have “recovered.” Furthermore, the significant time-by-group interaction effect observed in RMSSD and VAS measurements in our study reflects the prolonged impact of weight-cutting on both physiological recovery and psychological readiness. This aligns with Koral and Dosseville (2009) findings, where rapid and substantial weight loss before competitions led to increased confusion and tension, as well as reduced vigor, even after rehydration and nutrient intake (Koral and Dosseville, 2009). In contrast, the control group's relatively stable RMSSD and VAS values suggest that athletes who avoid aggressive weight-cutting practices may benefit from more consistent autonomic regulation and psychological stability leading into competition. In the present study, HRV was assessed using an ECG-based chest-strap monitor, allowing reliable tracking of autonomic changes across the weight-cutting and competition period. The use of a validated chest-strap system ensured accurate RMSSD quantification, which is particularly important when examining subtle alterations in autonomic regulation associated with rapid weight loss (Kim et al., 2018; Velmovitsky et al., 2022).

### Strengths and limitations of the study

This study has several strengths, including its real-world application and the use of a chest-strap ECG-based wearable sensor to monitor HRV across multiple time points in elite athletes during a critical competitive period. The integration of both objective (RMSSD) and subjective (VAS) stress metrics provides a comprehensive view of the physiological and psychological impacts of weight-cutting on athletes. The use of a validated electrode-based device (Polar H10) enabled accurate, non-invasive, and competition-compatible data collection, allowing HRV monitoring without disrupting athletes' routines. However, the study also has limitations. While chest-strap ECG-based sensors provide reliable HRV measurements in applied sports settings, they do not fully match the precision of multi-lead clinical ECG systems, which remain the gold standard for autonomic assessment. In addition, the study included a relatively small sample of male elite kickboxers, which may limit the generalizability of the findings to female athletes, non-elite populations, or other combat sports. Finally, despite efforts to standardize measurement conditions, uncontrolled environmental and psychological factors inherent to real competition settings may have influenced HRV and stress responses.

## Conclusion

Athletes in the weight-cutting group consistently exhibited lower RMSSD values and higher VAS scores compared to the control group across multiple measurement points, indicating elevated physiological and subjective stress throughout the weight-cutting process. Stress levels increased markedly before the weigh-in and remained higher than control levels during the pre-competition period. Although subjective stress decreased immediately after the weigh-in and continued to decline toward match day, RMSSD values showed that autonomic recovery lagged behind perceived recovery. This divergence suggests that weight-cutting athletes may regain a sense of readiness before competition despite incomplete physiological restoration. In contrast, the control group demonstrated more stable RMSSD and VAS profiles, with smaller fluctuations across time points. The between-group differences observed in both objective and subjective measures reflect the cumulative strain imposed by rapid weight reduction.

From a practical perspective, these findings highlight the importance of monitoring both autonomic and perceptual stress markers during the competition week, particularly from the weigh-in morning through match day. Coaches and medical staff should be cautious when relying solely on subjective indicators of recovery, as improvements in perceived readiness may mask ongoing autonomic strain. Integrating RMSSD monitoring alongside VAS assessments may support more informed decisions regarding rehydration strategies, recovery timing, and readiness to compete following rapid weight loss.

Overall, the findings indicate that rapid weight-cutting is associated with sustained autonomic stress and delayed physiological recovery, even when athletes report feeling subjectively improved. Monitoring both RMSSD and perceived stress may therefore provide a more comprehensive understanding of athlete readiness during periods of weight manipulation. Future studies should extend this work by including female athletes, athletes from different combat sports, and a broader range of weight categories to improve external validity and to better understand whether sex-, sport-, or weight-class–specific factors influence stress and recovery responses during weight-cutting.

## Data Availability

The data that support the findings of this study are available from the corresponding author, upon reasonable request. Requests to access the datasets should be directed to Süleyman Ulupinar, suleyman.ulupinar@erzurum.edu.tr.
